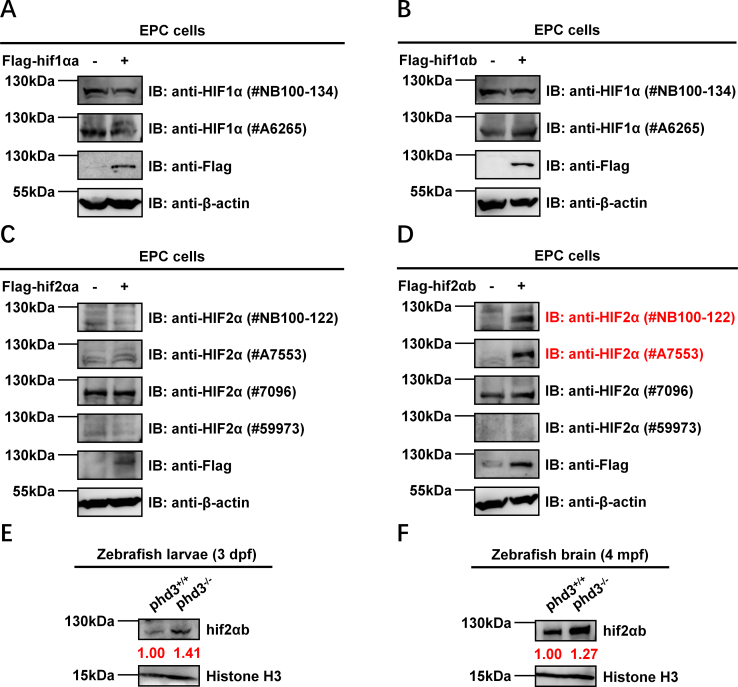# Deletion of prolyl hydroxylase domain-containing enzyme 3 (*phd3*) in zebrafish facilitates hypoxia tolerance

**DOI:** 10.1016/j.jbc.2023.105420

**Published:** 2023-11-03

**Authors:** Qian Liao, Hongyan Deng, Zixuan Wang, Guangqing Yu, Chunchun Zhu, Shuke Jia, Wen Liu, Yao Bai, Xueyi Sun, Xiaoyun Chen, Wuhan Xiao, Xing Liu

**Affiliations:** 1 State Key Laboratory of Freshwater Ecology and Biotechnology, Institute of Hydrobiology, Chinese Academy of Sciences, Wuhan, P. R.China; 2 Hubei Hongshan Laboratory, Wuhan, P. R.China; 3 University of Chinese Academy of Sciences, Beijing, P. R.China; 4 College of Life Science, Wuhan University, Wuhan, P. R.China; 5 The Innovation of Seed Design, Chinese Academy of Sciences, Wuhan, P. R.China

**Keywords:** phd3, hypoxia, zebrafish, hif-1α, hif-2α

## Abstract

Prolyl hydroxylase domain (PHD)-containing enzyme 3 (PHD3) belongs to the *Caenorhabditis elegans* gene *egl-9* family of prolyl hydroxylases. PHD3 catalyzes proline hydroxylation of hypoxia-inducible factor α (HIF-α) and promotes HIF-α proteasomal degradation through coordination with the pVHL complex under normoxic conditions. However, the relationship between PHD3 and the hypoxic response is not well understood. In this study, we used quantitative real-time PCR assay and *O*-dianisidine staining to characterize the hypoxic response in zebrafish deficient in *phd3*. We found that the hypoxia-responsive genes are upregulated and the number of erythrocytes was increased in *phd3*-null zebrafish compared with their wild-type siblings. On the other hand, we show overexpression of *phd3* suppresses HIF-transcriptional activation. In addition, we demonstrate phd3 promotes polyubiquitination of zebrafish hif-1/2α proteins, leading to their proteasomal degradation. Finally, we found that compared with wild-type zebrafish, *phd3*-null zebrafish are more resistant to hypoxia treatment. Therefore, we conclude phd3 has a role in hypoxia tolerance. These results highlight the importance of modulation of the hypoxia signaling pathway by phd3 in hypoxia adaptation.

Aerobic organism survival requires a constant supply of oxygen (O_2_) for the generation of ATP by oxidative phosphorylation (1, 2, 3, 4). Under low O_2_ (hypoxic) conditions, cells activate a number of adaptive responses to balance O_2_ supply and demand (4, 5, 6). In this process, hypoxia-inducible factors (HIFs) serve as master regulators to control the expression of thousands of genes for hypoxic adaptation (4). The PHD family of oxygen-dependent prolyl hydroxylases, including PHD1, PHD2, and PHD3 (as known as EGLN2, EGLN1 and EGLN3), plays a pivotal role in regulating HIF stability (3, 4, 7). Under normoxic conditions, HIF-1/2α are hydroxylated by PHDs and subsequently degraded by the von Hippel-Lindau (VHL) protein complex through proteasomal degradation, leading to the downregulation of the hypoxia signaling pathway (1, 3). Although all three members of the PHD family can hydroxylate HIF-1/2α *in vitro*, PHD2 seems to be the primary HIF-1/2α prolyl hydroxylase, as elimination of PHD2 alone is sufficient to robustly induce HIF-1/2α, and disruption of *Phd2* in mice causes embryonic lethality, similar to germline *Vhl* inactivation, whereas embryos lacking *Phd1* and *Phd3* are viable (8, 9, 10, 11, 12, 13).

Noteworthy, PHD3 is a HIF target gene and is markedly induced by hypoxia, suggesting that PHD3 may participate in a feedback loop to suppress HIF response (8, 12, 13, 14, 15). However, organisms with evolutionary adaptation to living at high altitudes (hypoxia) usually exert genetic variants in the gene encoding PHD2 (15). In addition, somatic inactivation of *Phd2*, but not *Phd3*, causes the development of polycythemia due to excessive production of erythropoietin, a hypoxia-responsive gene (12, 16, 17, 18). Moreover, hypomorphic germline *PHD2* mutations are associated with polycythemia in humans (15, 19, 20, 21). Therefore, although PHD3 belongs to the PHD family and negatively regulates HIF-1/2α like PHD2, whether PHD3 is involved in hypoxia adaptation or hypoxia tolerance has remained a mystery.

Zebrafish have evolutionarily conserved PHD family members, including *phd1, phd2a, phd2b*, and *phd3*, whose functions might be similar to those of mammalian PHDs (22, 23). Different from terrestrial organisms, fish encounter more frequent fluctuations of oxygen and they are often threatened by low oxygen levels in the water (24, 25). Therefore, zebrafish might be a good model for exploring the role of PHDs in hypoxia tolerance as previous similar studies have shown (26, 27, 28, 29). Interesting, the zebrafish embryos with mutated *vhl* exhibit a systemic hypoxic response with upregulation of the hypoxia-responsive genes and display typical features of the human VHL-associated disorder, indicating a similar function between mammalian *VHL* and zebrafish *vhl* (30). Furthermore, *phd3* is one of the most highly expressed genes in *vhl* mutants at various stages compared with that in the wildtype zebrafish (31, 32), further suggesting that zebrafish might be an ideal model for investigating the role of *phd3* in hypoxia tolerance.

Zebrafish have two orthologous copies of each *hif* gene, *hif1αa* and *hif1αb, hif2αa* and *hif2αb* (33). In this study, we find that the expression of *phd3* can only be induced by hif1αb, hif2αa, and hif2αb. Moreover, hypoxia-responsive genes are upregulated, and the number of erythrocytes is increased in *phd3*-null zebrafish compared with those in their wildtype siblings, leading to higher hypoxia tolerance in *phd3*-null zebrafish. Further assays indicate that phd3 suppresses HIF transcriptional activation. In addition, phd3 promotes polyubiquitination of hif-1/2α, thereby inducing proteasomal degradation of hif-1/2α. This study reveals the role of phd3 in hypoxia tolerance, highlighting the importance of modulation of the hypoxia signaling pathway by phd3 in hypoxia adaptation.

## Results

### Zebrafish *phd3* is induced under hypoxia

**Figure 1:**
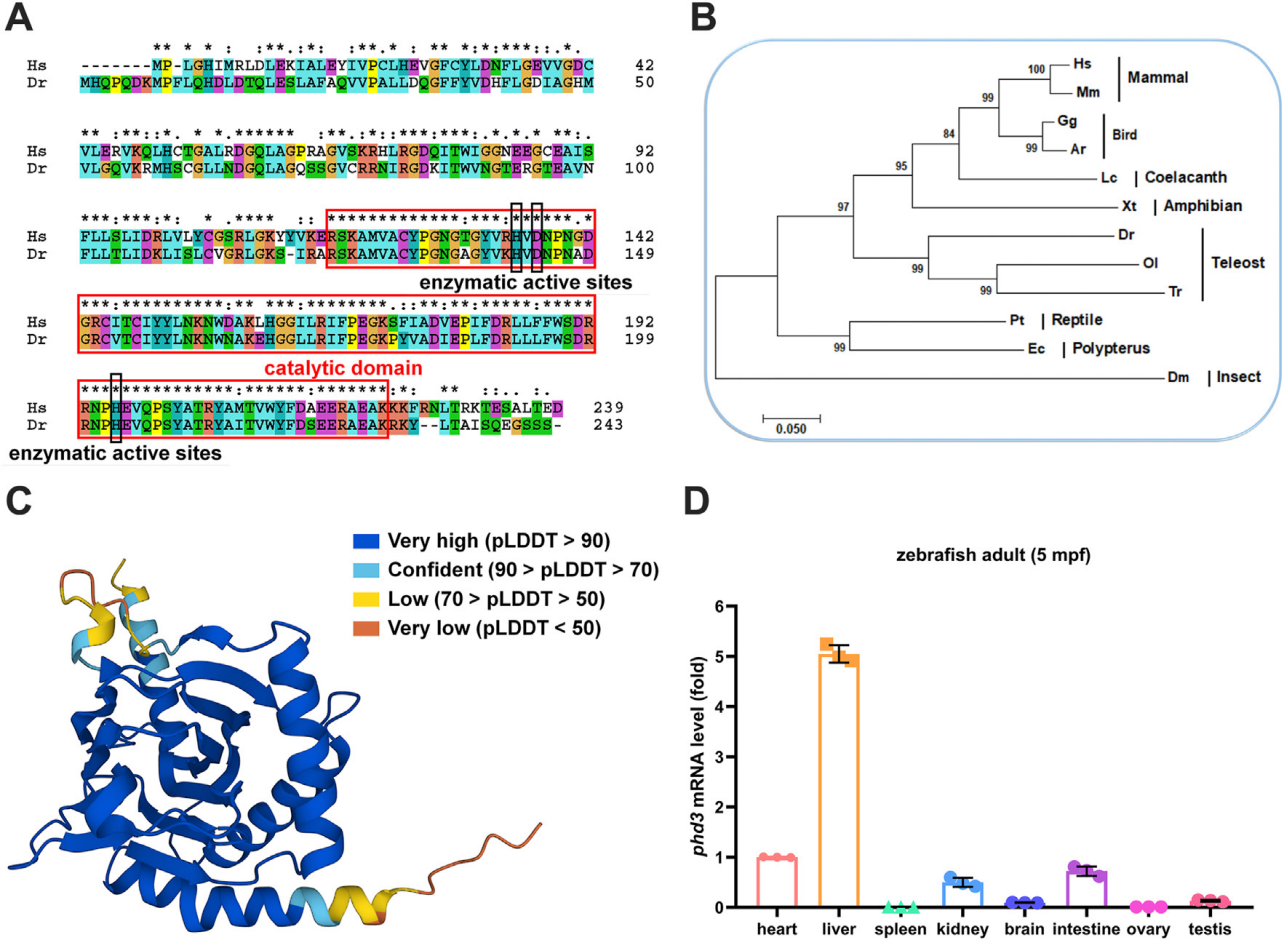
**Phylogenetic analysis of PHD3 proteins and the expression pattern of zebrafish *phd3* in different tissues.***A*, alignment of PHD3 amino acid sequences of *Homo sapiens* (*Hs*) and *Danio rerio* (*Dr*). *B*, the phylogenetic tree of PHD3 proteins from 12 species was constructed using the neighbor-joining method. *Homo sapiens* (*Hs*), NM_022073.4; *Mus musculus* (*Mm*), NM_028133.2; *Gallus gallus* (*Gg*), XM_421233.6; *Apteryx rowi* (*Ar*), XM_026085427.1; *Latimeria chalumnae* (*Lc*), XM_005989196.2; *Xenopus tropicalis* (*Xt*), NM_001127012.1; *Danio rerio* (*Dr*), NM_213310.1; *Oryzias latipes* (*Ol*), XM_023951862.1; *Takifugu rubripes* (*Tr*), XM_029833019.1; *Pseudonajia textilis* (*Pt*), XM_026710948.1; *Erpetoichthys calabaricus* (*Ec*), XM_028821616.1; and *Drosophila melanogaster* (*Dm*), NM_001300242.1. *C*, the predicted protein structure of zebrafish phd3 by AlphaFold in Ensembl database (https://www.asia.ensembl.org/index.html). *D*, quantitative real-time PCR (qPCR) analysis of *phd3* mRNA levels in different tissues of adult zebrafish (5 months post-fertilization, mpf).

Given that *phd3* is one of the most highly hypoxia-inducible genes in zebrafish and zebrafish is suitable for investigating hypoxia tolerance (26, 30, 31, 33, 34, 35), we sought to know whether zebrafish phd3 has an impact on hypoxia tolerance and to understand the underlying mechanisms. Initially, we compared the homology between the human PHD3 protein and the zebrafish phd3 protein. Overall, PHD3 is evolutionarily conserved, and its catalytic domain shares 85.85% identity between human PHD3 and zebrafish phd3 (Fig. 1*A*). Phylogenetic analysis indicated that zebrafish phd3 is close to those of teleost fish (Fig. 1*B*). Structural prediction also showed that the main structure of PHD3 is conserved between human PHD3 and zebrafish phd3 (Fig. 1*C*) (36). Among 8 zebrafish tissues, *phd3* is highly expressed in the liver (Fig. 1*D*). These data suggest that *phd3* might have conserved functions.

**Figure 2:**
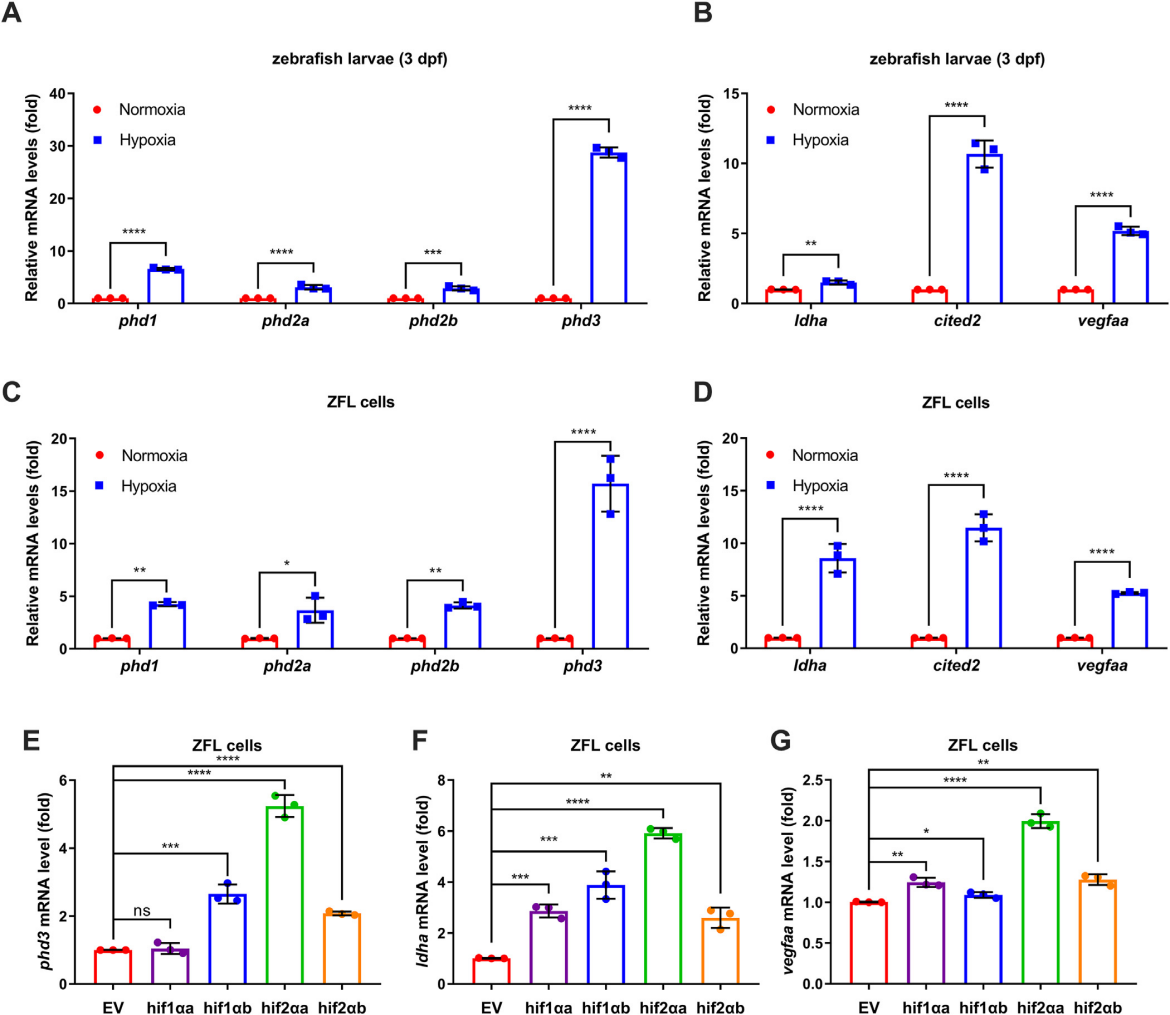
**Zebrafish *phd3* is induced under hypoxia.***A*, qPCR analysis of the prolyl hydroxylase genes (PHDs), including *phd1*, *phd2a*, *phd2b*, and *phd3* mRNA in zebrafish larvae (3 days post-fertilization, dpf) under normoxia (21% O_2_) or hypoxia (2% O_2_) for 5 h. *B*, qPCR analysis of hypoxia-responsive genes in zebrafish larvae (3 dpf), including *ldha*, *cited2*, and *vegfaa* under normoxia (21% O_2_) or hypoxia (2% O_2_) for 5 h. *C*, qPCR analysis of the PHD genes in ZFL cells, including *phd1*, *phd2a*, *phd2b*, and *phd3* under normoxia (21% O_2_) or hypoxia (1% O_2_) for 24 h. *D*, qPCR analysis of hypoxia-responsive genes in ZFL cells, including *ldha*, *cited2*, and *vegfaa* under normoxia (21% O_2_) or hypoxia (1% O_2_) for 24 h. *E*–*G*, qPCR analysis of *phd3* (*E*), *ldha* (*F*), and *vegfaa* (*G*) in ZFL cells transfected with Flag empty, Flag-*hif1αa*, Flag-*hif1αb*, Flag-*hif2αa*, or Flag-*hif2αb* for 24 h. ns, not significant; ∗*p* < 0.05, ∗∗*p* < 0.01, ∗∗∗*p* < 0.001, ∗∗∗∗*p* < 0.0001, using unpaired Student's *t* test; Data are representative of three independent experiments (mean ± SD of three technical replicates).

Next, we examined the expression of *phd3* in response to hypoxia. In zebrafish larvae, upon hypoxia treatment, *phd3* was induced at the highest level among 4 *phds* and typical hypoxia-responsive genes examined (Fig. 2, *A* and *B*). Similar results were obtained in ZFL cells (Fig. 2, *C* and *D*). Consistently, in ZFL cells, among four zebrafish *hif-1/2α* homologous genes, *phd3* was transactivated by ectopic expression of *hif1αb*, *hif2αa* and *hif2αb*, but not *hif1αa*, which is similar but not the same as *ldha* and *vegfaa*, two typical hypoxia-responsive genes (Fig. 2, *E*–*G*) (33). These data suggest that *phd3* is one of the most highly induced genes by hypoxia.

### Disruption of *phd3* in zebrafish enhances the expression of hypoxia-responsive genes and increases the number of erythrocytes in response to hypoxia

**Figure 3:**
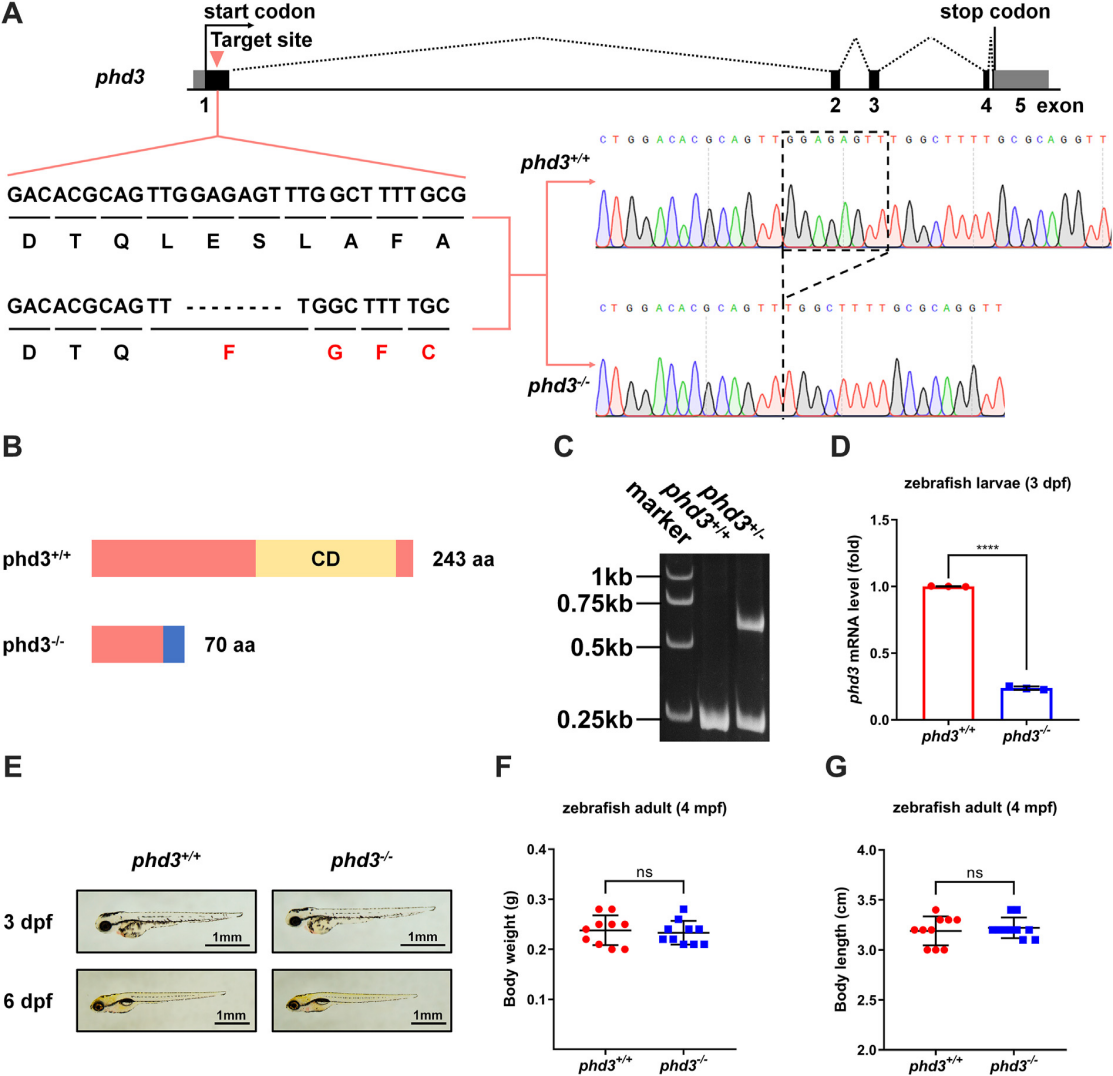
**Generation of *phd3*-null zebrafish and phenotype analysis.***A*, schematic of the sequence information of *phd3*-null zebrafish. 8-bp nucleotides (5′-GGAGAGTT-3′) were deleted in exon 1 of *phd3*-null mutant, resulting in a reading frame shift. *B*, the predicted protein products in the mutants (70 aa) and their wildtype siblings (243 aa). aa, amino acids. *C*, verification of CRISPR/Cas9-mediated zebrafish *phd3* disruption by heteroduplex mobility assay (HMA). *D*, qPCR analysis of *phd3* mRNA in *phd3*-null zebrafish larvae and their wildtype siblings (*phd3*^*−/−*^ or *phd3*^*+/+*^) (3 dpf). *E*, photographs of *phd3*-null zebrafish larvae and their wildtype siblings (*phd3*^*−/−*^ or *phd3*^*+/+*^) (3 dpf or 6 dpf). Scale bar = 1 mm. *F*, measurement of body weight of *phd3*-null adult zebrafish and their wildtype siblings (*phd3*^*−/−*^ or *phd3*^*+/+*^) (4 mpf). *G*, measurement of body length of *phd3*-null adult zebrafish and their wildtype siblings (*phd3*^*−/−*^ or *phd3*^*+/+*^) (4 mpf). ns, not significant; ∗∗∗∗*p* < 0.0001, using unpaired Student's *t* test; data are representative of three independent experiments (mean ± SD of three technical replicates).

**Figure 4:**
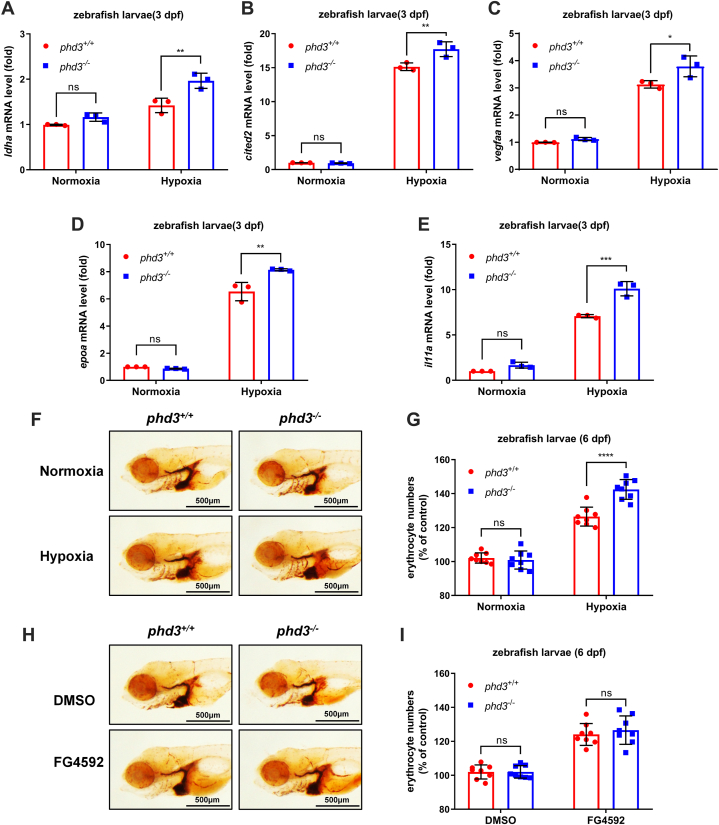
**Disruption of *phd3* in zebrafish enhances the expression of hypoxia-responsive genes and increase the number of erythrocytes in response to hypoxia.***A*–*E*, qPCR analysis of *ldha* (*A*), *cited2* (*B*), *vegfaa* (*C*), *epoa* (*D*), and *il11a* (*E*) mRNA levels in *phd3*-null zebrafish larvae and their wildtype siblings (*phd3*^*−/−*^ or *phd3*^*+/+*^) (3 dpf) under normoxia (21% O_2_) or hypoxia (2% O_2_) for 5 h. *F* and *G*, *O*-dianisidine staining of erythrocytes in *phd3*-null zebrafish larvae and their wildtype siblings (*phd3*^*−/−*^ or *phd3*^*+/+*^) (6 dpf) under normoxia (21% O_2_) or hypoxia (10% O_2_) for 12 h. The quantitation of erythrocytes in (*F*) was determined by normalizing the intensities of *O*-dianisidine stained cells. *H* and *I*, O-dianisidine staining of erythrocytes in *phd3*-null zebrafish larvae and their wildtype siblings (*phd3*^*−/−*^ or *phd3*^*+/+*^) (6 dpf) treated with FG4592 (20 μM) or DMSO as control for 12 h. The quantitation of erythrocytes in (*H*) was determined by normalizing the intensities of *O*-dianisidine stained cells. ns, not significant; ∗*p* < 0.05, ∗∗*p* < 0.01, ∗∗∗*p* < 0.001, ∗∗∗∗*p* < 0.0001, using unpaired Student's *t* test; data are representative of three independent experiments (mean ± SD of three technical replicates).

**Supporting Figure S1:**
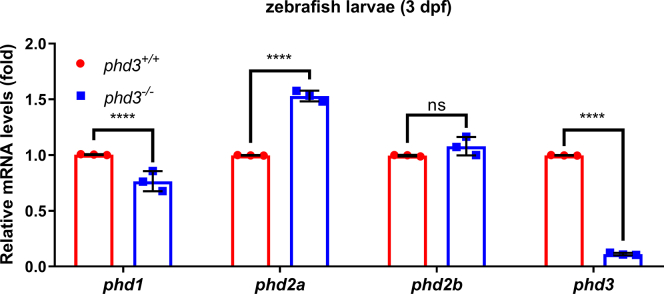


We then took advantage of *phd3*-null zebrafish to determine the effect of *phd3* on the hypoxia signaling pathway (22). Overall, no obvious phenotypes were observed in *phd3*^−/−^ zebrafish, including development, growth, reproduction, and body shape (Fig. 3, *A*–*G*). In addition, we examined the expression levels of *phd1*, *phd2a*, and *phd2b* in *phd3*^−/−^ zebrafish and found that only *phd2a* mRNA levels were slightly induced (Fig. S1). However, the expression of typical hypoxia-responsive genes, including *ldha, cited2, vegfaa, epoa,* and *il11a* (26, 33), were upregulated in *phd3*^−/−^ zebrafish larvae (3 dpf) compared with that in *phd3*^+/+^ zebrafish larvae under hypoxia (Fig. 4, *A*–*E*). Of note, under normoxia, the number of erythrocytes in *phd3*^−/−^ zebrafish larvae was pretty much the same as that in *phd3*^+/+^ zebrafish larvae (Fig. 4, *F* and *G*). But, under hypoxia, the number of erythrocytes in *phd3*^−/−^ zebrafish larvae was higher than that in *phd3*^+/+^ zebrafish larvae (Fig. 4, *F* and *G*). Moreover, after being treated with the PHD hydroxylase inhibitor, FG4592, the number of erythrocytes was also increased, but the number of erythrocytes in *phd3*^−/−^ zebrafish larvae showed no obvious difference compared with that in *phd3*^+/+^ zebrafish larvae upon FG4592 treatment (Fig. 4, *H* and *I*). Interestingly, the number of erythrocytes was still increased after *phd3*^−/−^ zebrafish were treated with FG4592 (Fig. 4*I*, column 4 *versus* column 2), indicating that other phd enzymes respond to FG4592 in the absence of phd3.

Taken together, these data suggest that disruption of *phd3* in zebrafish enhances the expression of hypoxia-responsive genes and increases the number of erythrocytes in response to hypoxia.

### Disruption of *phd3* in zebrafish facilitates hypoxia tolerance

**Figure 5:**
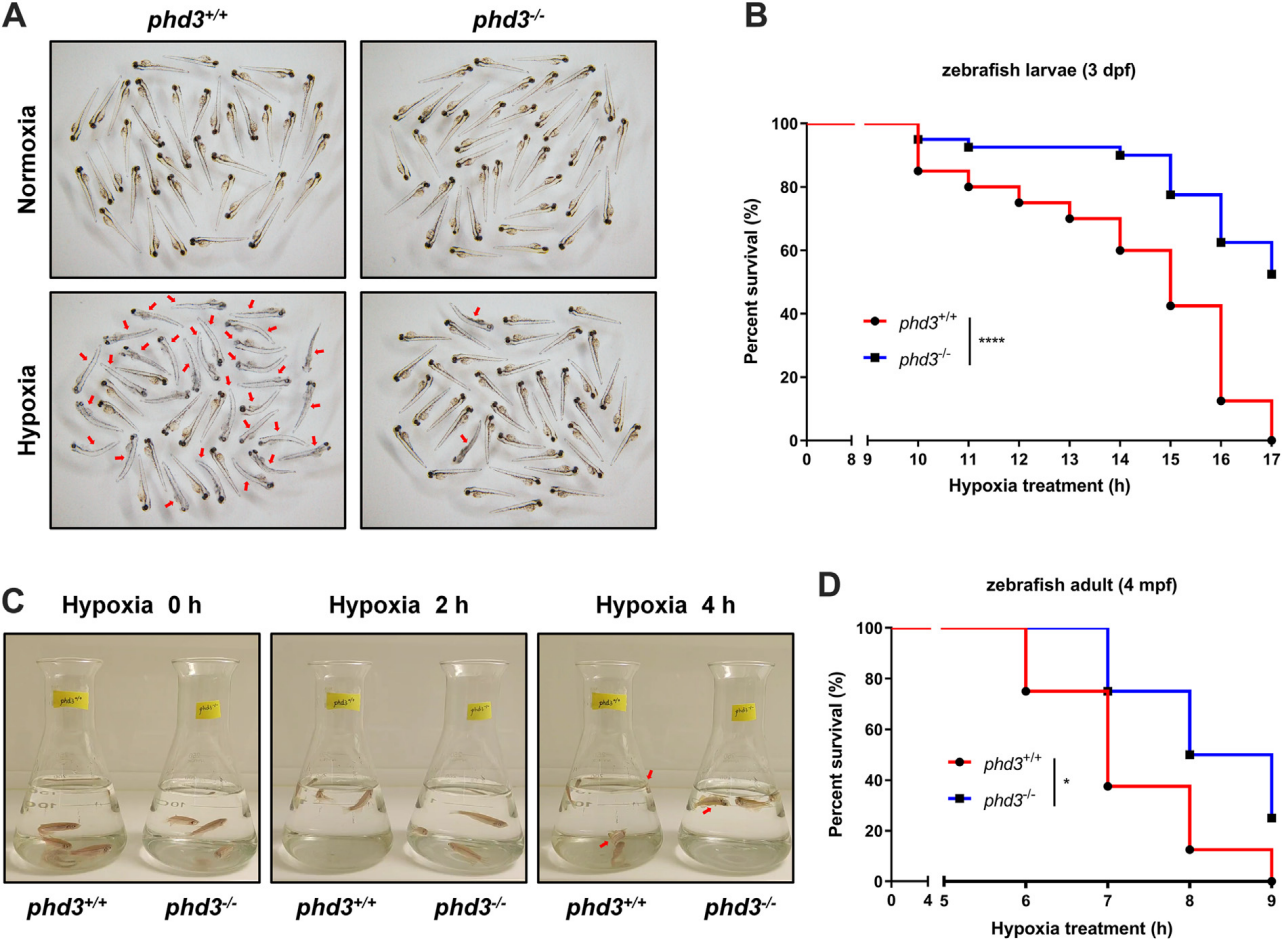
**Disruption of *phd3* in zebrafish facilitates hypoxia tolerance.***A*, photographs of *phd3*-null zebrafish larvae and their wildtype siblings (*phd3*^*−/−*^ or *phd3*^*+/+*^) (3 dpf) under normoxia (21% O_2_) or hypoxia (2% O_2_) for 15 h. *Red arrows* indicate dead zebrafish. *B*, survival curve of *phd3*-null zebrafish larvae and their wildtype siblings (*phd3*^*−/−*^ or *phd3*^*+/+*^) (3 dpf) under hypoxia (2% O_2_). *C*, photographs of *phd3*-null adult zebrafish and their wild-type siblings (*phd3*^*−/−*^ or *phd3*^*+/+*^) (4 mpf) under hypoxia (5% O_2_) for 0 h, 2 h, and 4 h. *Red arrows* indicate dead zebrafish. *D*, survival curve of *phd3*-null adult zebrafish and their wildtype siblings (*phd3*^*−/−*^ or *phd3*^*+/+*^) (4 mpf) under hypoxia (5% O_2_). ns, not significant; ∗*p* < 0.05, ∗∗∗∗*p* < 0.0001, using log-rank test analysis; data are representative of three independent experiments.

Given that the increase of the erythrocyte numbers in *phd3*-null zebrafish, we sought to know whether *phd3* has an impact on hypoxia tolerance. After *phd3*^+/+^ and *phd3*^−/−^ zebrafish larvae were simultaneously put into a hypoxia station (2% O_2_) for 15 h, more *phd3*^+/+^ zebrafish larvae displayed symptoms of death, such as no blood circulation, curvy body, and body degeneration, and so on. (Fig. 5, *A* and *B*). Moreover, when *phd3*^+/+^ and *phd3*^−/−^ adult zebrafish were put into the hypoxia station (5% O_2_), no difference in swimming behavior was observed between *phd3*^+/+^ and *phd3*^−/−^ zebrafish at the beginning (Fig. 5*C*, left panel). However, after 2 h, *phd3*^+/+^ zebrafish started to jump out of the water due to the intolerable low oxygen in the water, while *phd3*^−/−^ zebrafish still swam normally (Fig. 5*C*, middle panel). After 4 h, more *phd3*^+/+^ zebrafish died (Fig. 5*C*, right panel). The survival curve also indicated that *phd3*^−/−^ zebrafish were more resistant to hypoxic conditions.

Collectively, these data suggest that disruption of *phd3* in zebrafish facilitates hypoxia tolerance.

### Zebrafish phd3 suppresses HIF transcriptional activation

**Figure 6:**
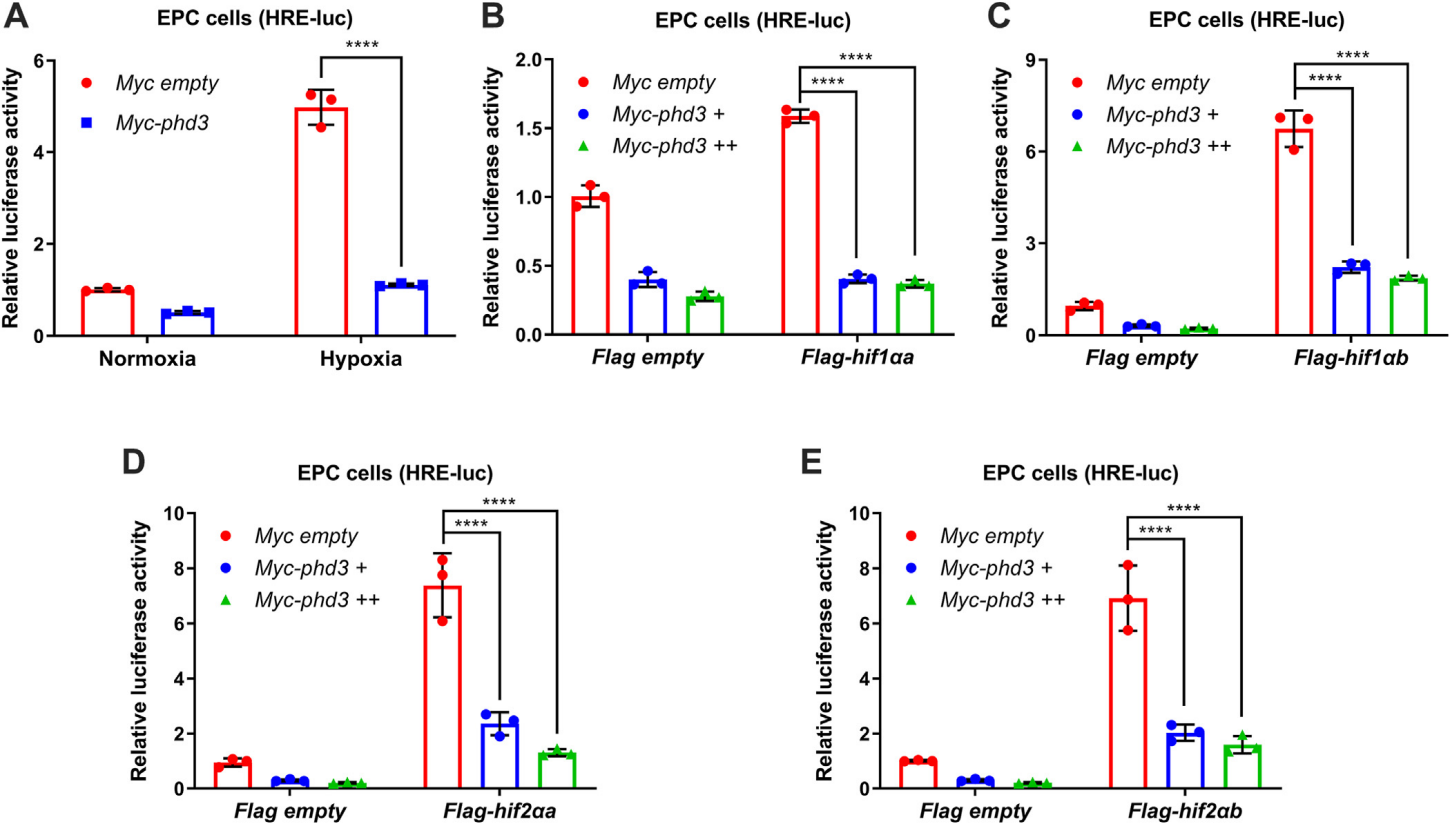
**Zebrafish phd3 suppresses HIF transcriptional activation.***A*, luciferase activity of hypoxia-responsive element (HRE) reporter in EPC cells transfected with Myc-*phd3* or Myc empty vector (control) under normoxia (21% O_2_) or hypoxia (1% O_2_) for 24 h. *B*, luciferase activity of the HRE reporter in EPC cells co-transfected with Flag-*hif1αa* or Flag empty vector (control) together with an increasing amount of Myc-*phd3* under normoxia (21% O_2_) for 24 h. *C*, luciferase activity of the HRE reporter in EPC cells co-transfected with Flag-*hif1αb* or Flag empty vector (control) together with an increasing amount of Myc-*phd3* under normoxia (21% O_2_) for 24 h. *D*, luciferase activity of the HRE reporter in EPC cells co-transfected with Flag-*hif2αa* or Flag empty vector (control) together with an increasing amount of Myc-*phd3* under normoxia (21% O_2_) for 24 h. *E*, luciferase activity of the HRE reporter in EPC cells co-transfected with Flag-*hif2αb* or Flag empty vector (control) together with an increasing amount of Myc-*phd3* under normoxia (21% O_2_) for 24 h. ns, not significant; ∗*p* < 0.05, ∗∗*p* < 0.01, ∗∗∗*p* < 0.001, ∗∗∗∗*p* < 0.0001, using two-way ANOVA analysis; data are representative of three independent experiments (mean ± SD of three technical replicates).

To validate whether zebrafish *phd3* functions like mammalian PHD3 in suppressing HIF transcriptional activation, we examined the effect of zebrafish phd3 on the activity of the hypoxia-responsive element (HRE)-driven luciferase reporter (33). As expected, overexpression of *phd3* significantly suppressed either hypoxia-induced HRE reporter activity, or hif1αa, hif1αb, hif2αa, or hif2αb-transactivated HRE reporter activity (Fig. 6, *A*–*E*). Therefore, zebrafish phd3 suppresses HIF transcriptional activation, functioning similar to mammalian PHD3.

### Zebrafish phd3 induces the degradation of hif-1/2α proteins by promoting the polyubiquitination of hif-1/2α

**Figure 7:**
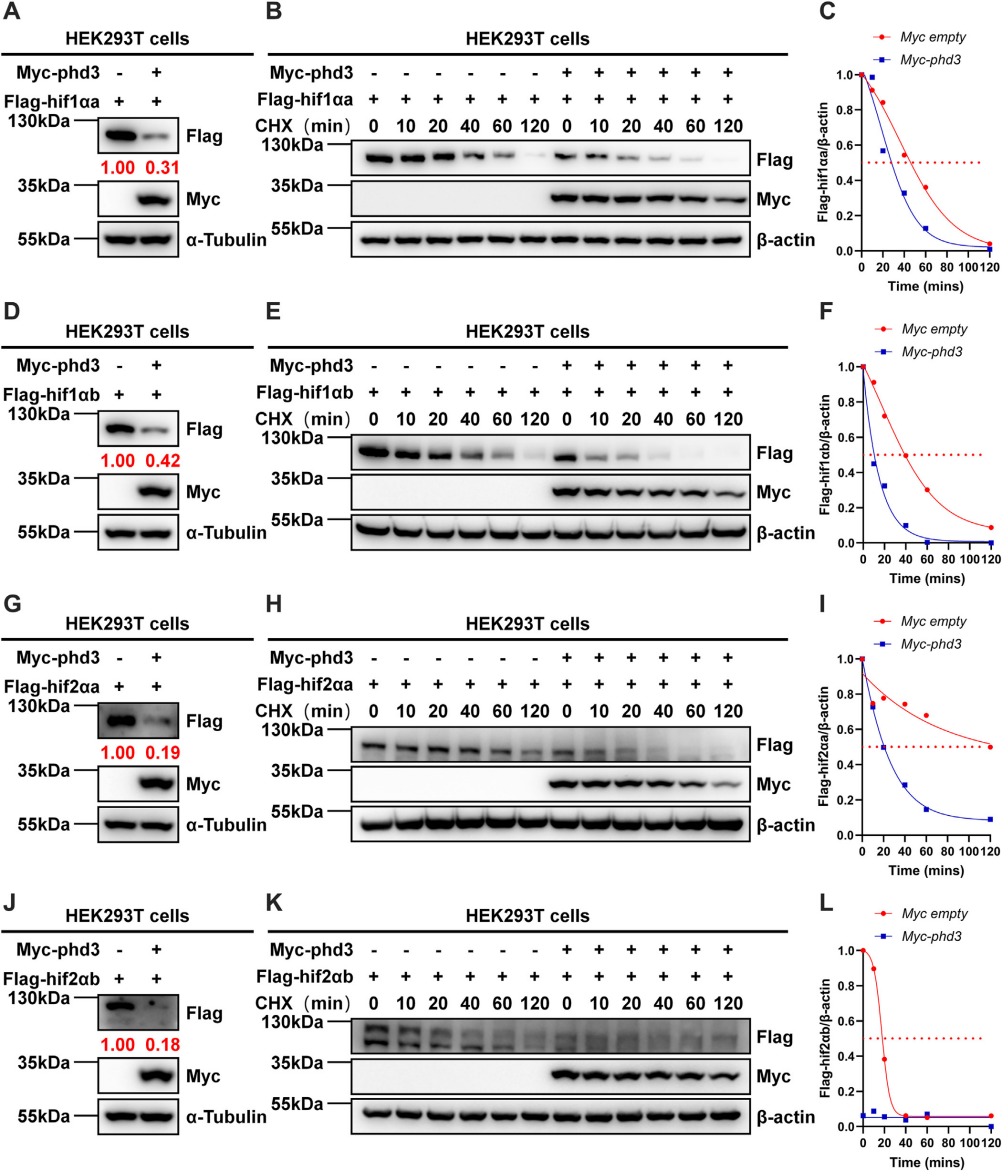
**Zebrafish phd3 induces the degradation of hif-1/2α proteins.***A*, Western blot analysis of hif1αa protein in HEK293T cells transfected with Flag-*hif1αa* together with Myc-*phd3* or Myc empty vector (control) for 24 h. *B* and *C*, Western blot analysis of hif1αa protein in HEK293T cells transfected with Flag-*hif1αa* together with Myc-*phd3* or Myc empty vector (control) for 20 h, followed by treatment with cycloheximide (CHX) (50 μg/ml) for the indicated time. The relative intensities of hif1αa in (*B*) were determined by normalizing the intensities of hif1αa to the intensities of β-actin. *D*, Western blot analysis of hif1αb protein in HEK293T cells transfected with Flag-*hif1αb* together with Myc-*phd3* or Myc empty vector (control) for 24 h. *E* and *F*, Western blot analysis of hif1αb protein in HEK293T cells transfected with Flag-*hif1αb* together with Myc-*phd3* or Myc empty vector (control) for 20 h, followed by treatment with CHX (50 μg/ml) for the indicated time. The relative intensities of hif1αb in (*E*) were determined by normalizing the intensities of hif1αb to the intensities of β-actin. *G*, Western blot analysis of hif2αa protein in HEK293T cells transfected with Flag-*hif2αa* together with Myc-*phd3* or Myc empty vector (control) for 24 h. *H* and *I*, Western blot analysis of hif2αa protein in HEK293T cells transfected with Flag-*hif2αa* together with Myc-*phd3* or Myc empty vector (control) for 20 h, followed by treatment with CHX (50 μg/ml) for the indicated time. The relative intensities of hif2αa in (*H*) were determined by normalizing the intensities of hif2αa to the intensities of β-actin. *J*, Western blot analysis of hif2αb protein in HEK293T cells transfected with Flag-*hif2αb* together with Myc-*phd3* or Myc empty vector (control) for 24 h. *K* and *L*, Western blot analysis of hif2αb protein in HEK293T cells transfected with Flag-*hif2αb* together with Myc-*phd3* or Myc empty vector (control) for 20 h, followed by treatment with CHX (50 μg/ml) for the indicated time. The relative intensities of hif2αb in (*K*) were determined by normalizing the intensities of hif2αb to the intensities of β-actin.

**Supporting Figure S2:**
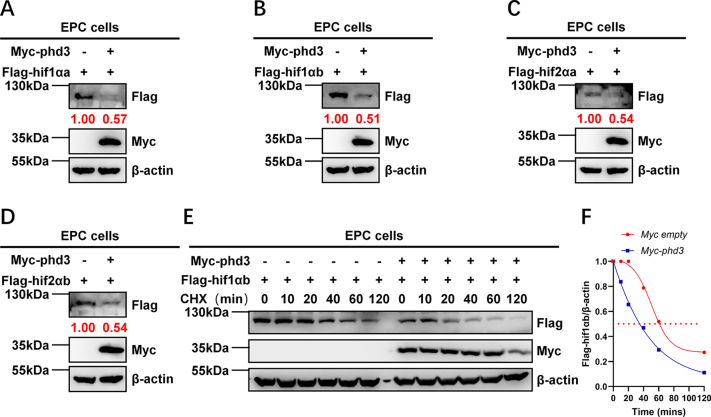


We subsequently intended to know whether zebrafish phd3 can mediate the degradation of zebrafish hif-1/2α proteins. As shown in Figure 7, overexpression of *phd3* clearly induced the downregulation of hif1αa, hif1αb, hif2αa, and hif2αb protein levels. The cycloheximide (CHX) treatment assays indicated that new protein synthesis was not required for phd3 to mediate the downregulation of hif-1/2α protein levels (Fig. 7, *A*–*L*), suggesting that phd3 may induce the degradation of hif-1/2α proteins. In addition, we confirmed these results in EPC cells (Fig. S2, *A*–*F*). Furthermore, we screened suitable antibodies that could detect endogenous zebrafish hif-α proteins (Fig. S3, *A*–*D*). Only two commercially available antibodies were able detect zebrafish hif2αb (Fig. S3*D*). As expected, hif2αb protein levels were higher in *phd3*-deficient zebrafish larvae or adult brains, compared with that in wild-type siblings (Fig. S3, *E* and *F*).

**Figure 8:**
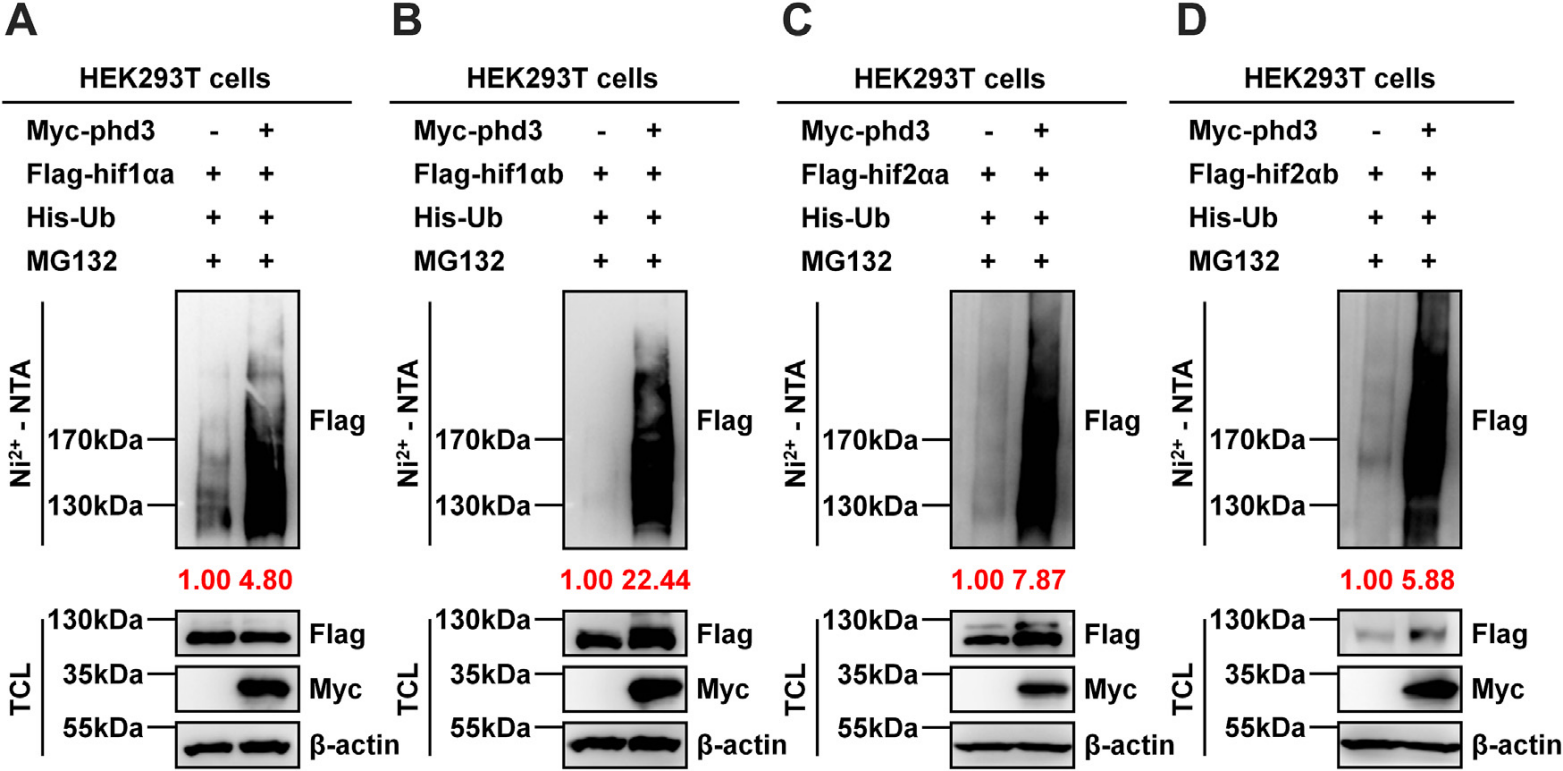
**Zebrafish phd3 promotes polyubiquitination of hif-1/2α proteins.***A*, the ubiquitination of hif1αa protein in HEK293T cells transfected with the indicated plasmids for 24 h, followed by MG-132 (20 μM) treatment for 8 h. The relative intensities of hif1αa ubiquitination were determined by normalizing the intensities of poly-ubiquitinated hif1αa in the Ni^2+^-pulldown to the intensities of hif1αa in the total cell lysates (TCL). *B*, the ubiquitination of hif1αb protein in HEK293T cells transfected with the indicated plasmids for 24 h, followed by MG-132 (20 μM) treatment for 8 h. The relative intensities of hif1αb ubiquitination were determined by normalizing the intensities of poly-ubiquitinated hif1αb in the Ni^2+^-pulldown to the intensities of hif1αb in the TCL. *C*, the ubiquitination of hif2αa protein in HEK293T cells transfected with the indicated plasmids for 24 h, followed by MG-132 (20 μM) treatment for 8 h. The relative intensities of hif2αa ubiquitination were determined by normalizing the intensities of poly-ubiquitinated hif2αa in the Ni^2+^-pulldown to the intensities of hif2αa in the TCL. *D*, the ubiquitination of hif2αb protein in HEK293T cells transfected with the indicated plasmids for 24 h, followed by MG-132 (20 μM) treatment for 8 h. The relative intensities of hif2αb ubiquitination were determined by normalizing the intensities of poly-ubiquitinated hif2αb in the Ni^2+^-pulldown to the intensities of hif2αb in the TCL.

Then, we examined whether zebrafish phd3 promotes polyubiquitination of hif-1/2α proteins. As expected, overexpression of *phd3* dramatically promoted the polyubiquitination of hif1αa, hif1αb, hif2αa, and hif2αb (Fig. 8, *A*–*D*).

These data suggest that zebrafish phd3 promotes polyubiquitination of zebrafish hif1αa, hif1αb, hif2αa, and hif2αb, thereby inducing their proteasomal degradation.

## Discussion

Hypoxia adaptation represents one of the most important physiological adaptations for organisms during the evolution process, such as for organisms living at high altitudes (37). These organisms have evolved the capability to tolerate much lower oxygen in the air than organisms living at lower altitudes. Although genetic variants associated with high-altitude adaptation in humans vary widely among populations (including Tibetan, Andean, and Ethiopian) (37, 38, 39), most terrestrial mammals living at high altitudes exhibit unique variants in the genes encoding HIF-2α and PHD2, two major components of the hypoxia signaling pathway (15, 37, 40, 41, 42, 43, 44). In this study, we found that disruption of *phd3* in zebrafish enhances the expression of hypoxia-responsive genes including *epoa*, which triggers an increase of erythrocytes in response to hypoxia. Erythrocytes are known to be responsible for O_2_ delivery (45). Thus, increased erythrocytes may enable organs and tissues to receive more oxygen, resulting in increased survival of zebrafish under hypoxia. This may be a possible mechanism accounting for the effect of disruption of *phd3* in zebrafish.

Interestingly, we found that overexpression of *phd3* strongly suppresses HIF-α transcriptional activation, but disruption of *phd3* only leads to moderately increased expression of hypoxia-inducible genes. In fact, HIF induces the expression of a range of downstream genes under hypoxic conditions. Although many of these genes are only moderately induced, this large number of induced genes work together to modulate the hypoxia response of the organism (46, 47, 48). In addition, we observed that *phd3* knockout could induce the expression of *phd2a*, which might compensate for the function of *phd3*. Therefore, the effect of *phd3* overexpression on the hypoxia signaling pathway is much stronger than that of *phd3* knockout. However, we identified that *phd3*-null zebrafish are more resistant to hypoxia treatment, highlighting the critical role of modulation of the hypoxia signaling pathway by phd3 in hypoxia tolerance. To further screen whether genetic variants of *PHD3* are presented in hypoxia-tolerant organisms will provide new molecular signatures for hypoxia adaptation.

Being different from terrestrial animals, fish live in water throughout their whole life span. Noteworthy, at the same altitude, compared with oxygen in air, oxygen dissolved in water is very unstable, which varies frequently depending on seasonal variation, the change of day and night, weather change, temperature change, depth of water, mobility of water, and organisms living in water, and so on. Consequently, different hypoxia-tolerant capabilities have been evolved by different fish species (25). This difference might cause some functional ambiguity of the hypoxia signaling pathway in hypoxia adaptation or tolerance between fish and terrestrial animals, such as the behavior of *phd3*. *Phd3* is one of the most highly induced genes by hypoxia in zebrafish (30, 31), but it is only mildly induced by hypoxia in mammalian cells (14). In addition, there are conflicting reports on the effect of PHD3 on HIF regulation, and mammalian PHD3 has been reported to be a transcriptional coactivator of HIF-1α instead of repressor (49, 50, 51). Thus, it is worth further characterizing whether *Phd3*-null mice also display hypoxia tolerance, which will give insight into the mechanistic difference in hypoxia tolerance between fish and terrestrial animals.

In the modern aquaculture industry, high-density aquaculture is becoming one of the major culture models. The oxygen concentration in water becomes a major limiting factor for fish live and growth in this kind of culture system (25, 52, 53). To resolve this issue, we need to cultivate fish strains with higher hypoxia tolerance. In this study, we found that the knockout of *phd3* in zebrafish does not affect fish development, growth, and reproduction but facilitates hypoxia tolerance. Our previous work also showed that the knockout of *phd3* in zebrafish benefits antiviral ability (22). Therefore, *phd3* appears to be an ideal target for cultivating fish strains with hypoxia tolerance as well as antiviral ability by genetic manipulation. Further identifying whether disruption of *phd3* in fish can affect other economic traits will be fully considered in order to breed better varieties in the aquaculture industry.

## Experimental procedures

### Sequence alignment and phylogenetic analysis

The amino acid sequences of phd3 from 12 indicated species were downloaded from the National Center for Biotechnology Information (NCBI) (https://www.ncbi.nlm.nih.gov) and subjected to alignment using the CLUSTAL W program. The phylogenetic tree based on the neighbor-joining method was constructed using MEGA7 software.

### Cell line and zebrafish

Zebrafish liver (ZFL) cells were originally obtained from the American Type Culture Collection (ATCC) and cultured in Ham’s F-12 medium (HyClone) supplemented with 10% fetal bovine serum (FBS) (Viva Cell). Epithelioma papulosum cyprini (EPC) cells (originally obtained from ATCC) were cultured in Medium 199 (Earle’s Salts Base) (Viva Cell) supplemented with 10% FBS. ZFL and EPC cells were maintained in a humidified incubator containing 5% CO_2_ at 28 °C. HEK293T cells (originally obtained from ATCC) were cultured in DMEM (VivaCell) supplemented with 10% FBS in a humidified incubator containing 5% CO_2_ at 37 °C. All cell lines were free from *mycoplasma* contamination.

Zebrafish (AB strain) were reared in a recirculating water system according to standard protocols. All zebrafish experiments were approved by the Institutional Animal Care and Use Committee of the Institute of Hydrobiology, Chinese Academy of Sciences.

### Generation of *phd3*-null zebrafish

The *phd3*-null zebrafish line, *phd3*^ihb^^q^^p3/ihb^^q^^p3^, was generated by CRISPR/Cas9-mediated mutagenesis. The procedure for generating *phd3*-null zebrafish has been described previously (22).

### Antibodies and chemical reagents

Anti-Myc antibody (#SC-40) was purchased from Santa Cruz Biotechnology. Anti-Flag antibody (#F1804) was purchased from Sigma-Aldrich. Anti-β-actin antibody (#AC026), anti-HIF-1α antibody (#A6265), and anti-HIF-2α antibody (#A7553) were purchased from ABclonal. Anti-HIF-1α antibody (#NB100-134) and anti-HIF-2α antibody (#NB100-122) were purchased from Novus. Anti-HIF-2α antibody (#7096) and anti-HIF-2α antibody (#59973) were purchased from Cell Signaling Technology. Anti-α-tubulin antibody (#62204) was purchased from Thermo Fisher Scientific. DMSO (#D2650) and MG-132 (#474790) were purchased from Sigma-Aldrich. FG4592 (#S1007) was purchased from Selleck. Cycloheximide (CHX) (#HY-12320) was purchased from MedChemExpress.

### *O*-dianisidine staining

The *phd3*-null zebrafish larvae [3 days post-fertilization (dpf); n = 10] and their wildtype siblings (3dpf; n = 10) in disposable 60-mm cell culture dishes filled with 5 ml egg water were incubated under normoxic and hypoxic conditions, or the indicated larvae were treated with FG4592 (20 μM) or DMSO as a control for 12 h. The larvae were then incubated with *O*-dianisidine solution (Sigma-Aldrich *O*-dianisidine dissolved in 100% ethanol, 0.1 M sodium acetate, and 30% H_2_O_2_) in a 12-well plate for 1 h. After incubation, the larvae were washed with ddH_2_O and fixed with 4% paraformaldehyde in PBS overnight at 4 °C. The larvae were then incubated in a bleaching solution (0.9% H_2_O_2_, 0.8% KOH, and 0.1% Tween in ddH_2_O) for 30 min to remove their natural pigmentation, and then in 4% paraformaldehyde for further fixation. After these steps, the larvae were immersed in 3% methylcellulose-M450 solution in a 100-mm cell culture dish and imaged using a Nikon TE2000-U microscope.

### Plasmid construction and transfection

Zebrafish *phd3* (Gene ID: ZDB-GENE-040426-2541) was amplified and subcloned into the pCMV-Myc vector. Zebrafish *hif1αa* (Gene ID: ZDB-GENE-080917-55), *hif1αb* (Gene ID: ZDB-GENE-040426-706), *hif2αa* (Gene ID: ZDB-GENE-030131-4490), and *hif2αb* (Gene ID: ZDB-GENE-060607-11) were amplified and subcloned into the pCMV-Flag vector. The accuracy of all constructs was confirmed by DNA sequencing. The plasmids were transfected into the indicated cells using VigoFect reagent (#T001; Vigorous Biotech, Beijing, China) according to the manufacturer’s instructions.

### Quantitative real-time PCR assay

RNAiso Plus (TaKaRa Bio) was used for total RNA extraction according to the manufacturer’s instructions. Revert Aid First Strand cDNA Synthesis Kit (Thermo Scientific) was used for cDNA synthesis. MonAmp SYBR Green qPCR Mix (high Rox) (Monad Bio.) was used for quantitative real-time PCR assays. Primers are listed in Table S1.

### Luciferase reporter assay

Cells seeded in 24-well plates were transfected with the indicated plasmids together with pCMV-Renilla as an internal control. Twenty-4 h after transfection, luciferase reporter activity was determined using the dual-luciferase reporter assay system (#E1960, Promega). Data were normalized to *Renilla* luciferase. Data are expressed as mean ± SD of a representative experiment performed in triplicate based on 3 independent experiments.

### Western blot analysis

Cells were washed with ice-cold PBS and then lysed in RIPA solution containing 150 mM NaCl, 50 mM Tris (pH 7.4), 1 mM NaF, 1 mM EDTA (pH 8.0), 1% Nonidet P-40, 0.25% sodium deoxycholate, 1 mM PMSF, 1 mM Na_3_VO_4_, and a 1:100 dilution of protease inhibitor mixture (Sigma-Aldrich) for 30 min at 4 °C. Cell lysates were separated by 10% SDS-PAGE, incubated with the indicated antibodies, and photographed on a Fuji Film LAS4000 mini-luminescence image analyzer using ECL Western 400 blotting detection reagent (Millipore). Image J software (National Institutes of Health) was used to quantify protein levels based on the band density obtained by Western blot analysis.

### Ubiquitination assay

HEK293T cells seeded in 100-mm cell culture dishes were co-transfected with His-*ubiquitin* (5 μg), Myc-*phd3* (3 μg) or Myc empty (3 μg) as control, together with Flag-*hif1αa* (3 μg) or Flag-*hif1αb* (3 μg) or Flag-*hif2αa* (3 μg) or Flag-*hif2αb* (3 μg), respectively, for 16 h. Cells were treated with MG-132 (20 μM) for 8 h and then lysed with denatured buffer (6M guanidine-HCl, 0.1 M Na_2_HPO_4_/NaH_2_PO_4_, 0.01 M imidazole), followed by Ni^2+^-NTA agarose purification and immunoblotting with the indicated antibodies.

### Statical analysis

GraphPad Prism software (8.3.0) was used for all statistical analyses. Results with error bars represent mean ± SD. Survival data were calculated by the Kaplan-Meier method and analyzed by the log-rank test. Other statistical analyses were performed using unpaired Student's *t* test or two-way ANOVA analysis. *p* values less than 0.05 were considered significant. Statistical significance is indicated as follows: ∗*p*< 0.05, ∗∗*p* < 0.01, ∗∗∗*p* < 0.001, ∗∗∗∗*p* < 0.0001; data are representative of three independent experiments performed in three technical repeats.

## Data availability

Further information and requests for resources and reagents should be directed to and will be fulfilled by Xing Liu and Wuhan Xiao.

## Supporting information

This article contains supporting information.

## Conflict of interest

The authors declare that they have no conflicts of interest with the contents of this article.

## Figures and Tables

**Supporting Figure S3:**