# Supplementary material for: Deletion of prolyl hydroxylase domain-containing enzyme 3 (phd3) in zebrafish facilitates hypoxia tolerance
Source: J Biol Chem. 2023 Nov 3;299(12):105420. doi: 10.1016/j.jbc.2023.105420 (PMC10724695; doi:10.1016/j.jbc.2023.105420)
Supplement: Supporting Figures S1–S3 and Table S1 [file mmc1.docx]

**Supplementary information**

**Supplementary Data**

**Fig. S1. Expression of different *phds* in wild-type and *phd3*-null zebrafish.**

qPCR analysis of *phd1*, *phd2a*, *phd2b*, and *phd3* mRNA levels in *phd3*-null zebrafish larvae and their wild-type siblings (*phd3^-/-^* or *phd3^+/+^*) (3 dpf). ns, not significant; ****p < 0.0001, using unpaired Student's t-test; data are representative of three independent experiments (mean ± SD of three technical replicates).

**Fig. S2. Zebrafish phd3 induces the degradation of hif-1/2α proteins in EPC cells.**

(A) Western blot analysis of hif1αa protein in EPC cells transfected with Flag-*hif1αa* together with Myc-*phd3* or Myc empty vector (control) for 24 h.

(B) Western blot analysis of hif1αb protein in EPC cells transfected with Flag-*hif1αb* together with Myc-*phd3* or Myc empty vector (control) for 24 h.

(C) Western blot analysis of hif2αa protein in EPC cells transfected with Flag-*hif2αa* together with Myc-*phd3* or Myc empty vector (control) for 24 h.

(D) Western blot analysis of hif2αb protein in EPC cells transfected with Flag-*hif2αb* together with Myc-*phd3* or Myc empty vector (control) for 24 h.

(E-F) Western blot analysis of hif1αb protein in EPC cells transfected with Flag-*hif1αb* together with Myc-*phd3* or Myc empty vector (control) for 20 h, followed by treatment with cycloheximide (CHX) (50 μg/ mL) for the indicated time. The relative intensities of hif1αb in (E) were determined by normalizing the intensities of hif1αb to the intensities of β-actin.

**Fig. S3.** **Disruption of *phd3* in zebrafish increases endogenous hif2αb protein levels.**

(A) Western blot analysis of overexpressed Flag-hif1αa protein in EPC cells with various anti-HIF-1α antibodies.

(A) Western blot analysis of overexpressed Flag-hif1αb protein in EPC cells with various anti-HIF-1α antibodies.

(C) Western blot analysis of overexpressed Flag-hif2αa protein in EPC cells with various anti-HIF-2α antibodies.

(D) Western blot analysis of overexpressed Flag-hif2αb protein in EPC cells with various anti-HIF-2α antibodies. The catalog number marked in red indicates that the antibody is working well.

(E) Western blot analysis of endogenous hif2αb protein levels with anti-HIF-2α antibody (#NB100-122) in *phd3*-null zebrafish larvae and their wild-type siblings (*phd3^-/-^* or *phd3^+/+^*) (3 dpf) under hypoxia (2% O_2_) for 4 hours.

(F) Western blot analysis of endogenous hif2αb protein levels with anti-HIF-2α antibody (#A7553) in the brains of *phd3*-null adult zebrafish and their wild-type siblings (*phd3^-/-^* or *phd3^+/+^*) (4 mpf) under hypoxia (5% O_2_) for 1 hour.

**Supplemental Table S1. The** **quantitative real-time PCR primer sequences**

| **Primers** | **Sequence (5’ to 3’)** |
| --- | --- |
| zebrafish actb1 (internal control) –RT  (Gene ID: ZDB-GENE-000329-1) | F: TACAATGAGCTCCGTGTTGC |
|  | R: ACATACATGGCAGGGGTGTT |
| zebrafish *phd1*-RT  (Gene ID: ZDB-GENE-060503-757) | F: GGGGATTGTGTCATCAATG |
|  | R: CTCCATGAACCTTCACATCC |
| zebrafish *phd2a*-RT  (Gene ID: ZDB-GENE-110408-34) | F: ATTCTGGTCTGACAGACG |
|  | R: TTCACCAGCACCTGTAAG |
| zebrafish *phd2b*-RT  (Gene ID: ZDB-GENE-040718-338) | F: ACAGCTGGTCAGTCAGAG |
|  | R: ATCAGATCGTCCATGCGG |
| zebrafish *phd3*-RT  (Gene ID: ZDB-GENE-040426-2541) | F: CGCTGCGTCACCTGTATT |
|  | R: TAGCATACGACGGCTGAACT |
| zebrafish *ldha*-RT  (Gene ID: ZDB-GENE-991026-5) | F: CCTTCTCAAGGATCTGACCG |
|  | R: ACACTGTAATCTTTATCCGC |
| zebrafish *cited2*-RT  (Gene ID: ZDB-GENE-041010-141) | F: GTTCCGAGACAGTATCGCTAAG |
|  | R: ATCAAGACCTCCTCGTCAATAA |
| zebrafish *vegfaa*-RT  (Gene ID: ZDB-GENE-990415-273) | F: TGCTCCTGCAAATTCACACAA |
|  | R: ATCTTGGCTTTTCACATCTGCAA |
| zebrafish *epoa*-RT  (Gene ID: ZDB-GENE-061218-3) | F: GAAGTCTGGGAAGCGATGAAT |
|  | R: CGGTATGCTGAGACTTCGCAG |
| zebrafish *il11a*-RT  (Gene ID: ZDB-GENE-051019-1) | F: CCGGGTGTTTAGTACAGAGATT |
|  | R: CATGGAGCTGAGAAAGAGTAGG |
